# Optimising Integrated Stroke Care in Regional Networks: A Nationwide Self-Assessment Study in 2012, 2015 and 2019

**DOI:** 10.5334/ijic.5611

**Published:** 2021-09-20

**Authors:** Helene R. Voogdt-Pruis, Nick Zonneveld, Monique Bergsma, Elize van Wijk, Henk Kerkhoff, Luikje van der Dussen, Maartje Kuijpens, Hubertus J. M. Vrijhoef, Mirella M. N. Minkman

**Affiliations:** 1 University Medical Centre Utrecht, Julius Global Health, Utrecht University, PO Box 85500, 3508 GA Utrecht, Netherlands; 2 Encorps, 1214 ND, Hilversum Netherlands; 3 Tilburg University, TIAS School for Business and Society, PO Box 90153, 5000 LE Tilburg, Netherlands; 4 Vilans, National Centre of Expertise in Long-term Care, PO Box 8228, 3503 RE Utrecht, Netherlands; 5 Stroke Knowledge Network Netherlands, Oudlaan 4, 3515 GA Utrecht, Netherlands; 6 Albert Schweitzer Hospital, Department of Neurology, PO Box 444, 3300 AK Dordrecht, Netherlands

**Keywords:** stroke services, integrated care, care coordination, collaboration, quality improvement, interprofessional teamwork, patient-centredness

## Abstract

**Background:**

To help enhance the quality of integrated stroke care delivery, regional stroke services networks in the Netherlands participated in a self-assessment study in 2012, 2015 and 2019.

**Methods:**

Coordinators of the regional stroke services networks filled out an online self-assessment questionnaire in 2012, 2015 and 2019. The questionnaire, which was based on the Development Model for Integrated Care, consisted of 97 questions in nine clusters (themes). Cluster scores were calculated as proportions of the activities implemented. Associations between clusters and features of stroke services were assessed by regression analysis.

**Results:**

The response rate varied from 93.1% (2012) to 85.5% (2019). Over the years, the regional stroke services networks increased in ‘size’: the median number of organisations involved and the volume of patients per network increased (7 and 499 in 2019, compared to 5 and 364 in 2012). At the same time, fewer coordinators were appointed for more than 1 day a week in 2019 (35.1%) compared to 2012 (45.9%). Between 2012 and 2019, there were statistically significantly more elements implemented in four out of nine clusters: ‘Transparent entrepreneurship’ (MD = 18.0% F(1) = 10.693, p = 0.001), ‘Roles and tasks’ (MD = 14.0% F(1) = 9.255, p = 0.003), ‘Patient-centeredness’ (MD = 12.9% F(1) = 9.255, p = 0.003), and ‘Commitment’ (MD = 11.2%, F(1) = 4.982, p = 0.028). A statistically significant positive correlation was found for all clusters between implementation of activities and age of the network. In addition, the number of involved organisations is associated with better execution of implemented activities for ‘Transparent entrepreneurship’, ‘Result-focused learning’ and ‘Quality of care’. Conversely, there are small but negative associations between the volume of patients and implementation rates for ‘Interprofessional teamwork’ and ‘Patient-centredness’.

**Conclusion:**

This long-term analyses of stroke service development in the Netherlands, showed that between 2012 and 2019, integrated care activities within the regional stroke networks increased. Experience in collaboration between organisations within a network benefits the uptake of integrated care activities.

## Background

Worldwide, stroke is the second leading cause of death and source of ‘disability adjusted life years’. The prevalence of stroke is expected to increase due to a growing and ageing population and due to lower stroke case fatality rates associated with better acute ischaemic stroke care and improved recurrent stroke prevention strategies addressing metabolic and behavioural factors [1]. Ischaemic stroke occurs when the blood supply to part of the brain is interrupted or reduced, preventing brain tissue from getting oxygen and nutrients. Less frequently, cerebral haemorrhages occur with similar, often devastating results. Early action and effective treatments can reduce brain damage, complications, and disabilities. Recovery after stroke mainly occurs within the first six months. Stroke patients can experience long-term difficulties in terms of quality of life, social reintegration, life satisfaction and emotional functioning, including depression and anxiety [2]. After a stroke, about 70 percent of patients are discharged back home from the hospital, about 20 percent are referred to rehabilitation centres or nursing homes and about 10 percent die within 30 days [3].

In order to deliver patient-centred integrated stroke care, cooperation and collaboration between professionals, patients and caregivers is of utmost importance. A seamless integration of services across the healthcare and social care interface is required for effective treatment and management of strokes [3]. In addition, integrated stroke care is needed to align healthcare services, to decrease repeated assessments and incomplete or conflicting information about the patient’s health status and to reduce duplications in supervision and multiple transaction costs [4]. However, various barriers could hinder the formation and development of such collaboration, for example related to existing administration and regulation, funding, organisational and interorganisational domains, service delivery and work routines [5]. From the patients’ perspective, quality of stroke care could be improved by better collaboration between professionals, by providing tailor-made information and by shared decision making [6,7]. In addition, better preparation of transitions from hospital to home care and tailored support at home is needed [3].

Integration of hospital and primary care services has been a priority of health policy in The Netherlands since the 1990s. Several initiatives focused on the implementation or the investigation of so called ‘transmural or integrated care’. Transmural care was defined as care attuned to the needs of the patient, provided on the basis of cooperation and coordination between general and specialised care professionals with shared overall responsibility and the specification of delegated responsibilities [8]. For stroke, first studies on the effectiveness of ‘transmural care’ in the Netherlands appeared early 2000. Reported benefits of ‘transmural stroke care’ six months after stroke were: higher patient satisfaction, higher portion of patients back home, and less volume of home care [9]. Further improvement of quality of ‘transmural or integrated stroke care’ was the aim of a national improvement project that started in 2002. Based on the Breakthrough Series [10] teams from 23 regions aimed to make ‘breakthrough’ improvements [11] on specific topics in stroke care. Significantly better results in health outcomes and interprofessional collaboration were attained [12]. The ‘sense of urgency’ for continuous improvement of integrated stroke care was set. Following this, more registered integrated stroke services emerged. Regional networks of service providers, such as hospitals, geriatric and medical rehabilitation centres, skilled nursing facilities, and primary care providers started to work together in an organised way [13]. These networks were led by a steering board with representatives —professionals and managers— of involved organisations. Needed were professionals and managers with the ability to create collaboration and cooperation across professions and organisations, that are comfortable with distributing responsibilities, and that thoroughly understand the aim of integrated stroke services [14]. Often one professional within the stroke service was appointed as coordinator. The aim was to work together to provide multidisciplinary, coordinated care through organised patient transfers and multi-disciplinary protocols. In addition, the ambition was to make continuous improvements on collaboration and cooperation of stroke care. Integrated stroke services in the Netherlands turned out to increase patient satisfaction and the portion of patients back home, as well as reducing the volume of home care [6,11,12,13,14].

In 2006, the Dutch Knowledge network of stroke services (KNCN) was founded to support all stroke services in the Netherlands in their mission to improve the coordination, cooperation and quality of regional multidisciplinary integrated stroke care. In 2006, 21 stroke services participated; this increased to 44 in 2009. In 2012 and 2019, 72 and 52 stroke services were member of KNCN. A decline in participating stroke services took place after 2014 due to the changed role of KNCN in the national Benchmark stroke care and discontinuation of financial support, and due to the merging of some stroke services. Nonetheless, in 2019, about 90% of all hospitals participated with their regional stroke service in KNCN. Most coordinators of the regional stroke services have a background as specialised stroke nurse and are appointed at the hospital. These coordinators participate in the national learning network of KNCN. Important skills of coordinators include: networking, organising meetings with professionals and organisations, supporting the exchange and/or exchanging (new) knowledge, collecting data on quality of care, promoting and/or making agreements on collaboration, reflecting on current practice and stimulating continuous quality improvement. Within the learning network, coordinators and KNCN identify and share ‘good practices’, guidelines and studies on specific topics in (integrated) stroke care. An online toolbox with this information and national meetings are available for all coordinators. An online forum facilitated communication between coordinators. In addition, educational courses on specific topics, such as ‘creating good collaboration within the stroke services’ are organised.

With the objective of encouraging continuous quality improvement, stroke services can participate in KNCNs self-assessment study to get insights into their own performance compared with earlier measuring moments and other stroke services. In the current study, we explored the results of KNCNs self-assessment studies in 2012, 2015 and 2019. The primary objective of our study was to establish which integrated care activities were implemented within the Dutch stroke services in 2012, 2015 and 2019. In addition, since a knowledge gap on the best size or scale for the governance of integrated stroke services exists [15], we explored whether implementation of integrated care activities was associated with characteristics of the stroke services involved in order to get more insight what organisational features might benefit or hamper the coordination and collaboration of integrated stroke care. A deeper insight in these relations are relevant; some studies indicate a relationship between the quality of integrated care networks and the volume of target group and/or the amount of collaborative partners [15].

## Methods

### Study design

All stroke services networks participating in KNCN, 72 (2012), 59 (2015) and 55 (2019), were invited for the study. Although some stroke services (e)merged during the years, our study could be regarded as a cohort study. Coordinators of all stroke services networks were invited to fill out an online self-assessment questionnaire on behalf of the stroke services network. Coordinators could discuss the questions with stakeholders or complete the questionnaire by themselves. This was up to the coordinators. Participation was voluntary. A short instruction about the self-evaluation tool was provided. The invitations were sent in February/March of the respective years; a reminder was sent to coordinators who did not respond initially. Data collection ended in April. Ethical approval from a medical ethics committee was not needed under Dutch law as this was an observational study among care professionals only.

### Self-assessment questionnaire

Coordinators were requested to provide general characteristics about their regional stroke services network and about implemented integrated care activities within the network. The Development Model for Integrated Care (DMIC) [16,17] served as a basis for the self-assessment questionnaire (in Dutch). The same questionnaire was used in all three years. After a brief explanation, the coordinators were asked to answer 97 questions about activities related to integrated stroke care, answering “yes”, “no” or “don’t know” (see Appendix 1). The DMIC is a validated model and consists of 97 activities [16,17] grouped into nine clusters:

1. Interprofessional teamwork: professionals collaborating and working in well-organised multidisciplinary teams in the care network to provide care for a well-described client group (3 items);
2. Roles and tasks: clarity about the expertise, roles and tasks of professionals in the stroke service with effective collaboration at all levels, with new partners and by allocating coordinating roles (8 items);
3. Patient-centeredness: integrated care and information flows are tailored to the needs and characteristics of patients. This also includes self-management support (10 items);
4. Commitment: collaborative commitment and goals in the care chain with clearly defined goals and collaborative targets (12 items);
5. Transparent entrepreneurship: conditions for innovation and leadership responsibilities for performance achievement and joint financial agreements covering the integrated care (7 items);
6. Result-focused learning: a learning climate aiming for continuous improvement in the results of the stroke service (14 items);
7. Quality of care: the design of a multidisciplinary care pathway throughout the care service, based on guidelines and with patient representatives’ involvement (including clients’ needs assessment) (7 items);
8. Delivery system: logistics and coordination procedures for streamlining the care process within the stroke service (e.g. agreements, procedures and tools) (18 items);
9. Performance management: measurement and analyses of the results of the care as delivered (performance targets, quality indicators, analysis of mistakes and near misses, feedback, improvement activities) (18 items).

Furthermore, the DMIC distinguishes four phases of development: (1) the initiative and design phase; (2) the experimental and execution phase; (3) the expansion and monitoring phase; and (4) the consolidation and transformation phase. Each of development is described and characterised by the ten most phase-relevant activities. The development phase of a network is assessed by the overall description and the presence of activities measured by the DMIC. A phase is considered implemented if at least seven out of ten phase specific items are met. (see Appendix 2).

### Data analyses

Descriptive analyses were conducted to study the characteristics of integrated stroke care services and the implementation of integrated stroke care activities. Stroke service cluster scores were calculated as the proportion (completed = ‘yes’) of implemented elements per cluster among all stroke services. The reported ‘don’t know’ answers for implemented elements were regarded as ‘not completed’, as coordinators filled out the questionnaire in collaboration with stakeholders of the regional network. The associations between the cluster scores and characteristics of services were examined by means of linear regression analysis. Stepwise forward selection (probability-of-F-to-enter <= 0.05) was used in order to analyse the association of characteristics and the clusters cautiously as we had no theoretical assumptions or model for the associations between characteristics and clusters. SPSS 25.0 was used for the data analyses.

## Results

### Respondents

Totals of 67 (2012), 53 (2015) and 47 (2019) stroke services responded to the invitation for the study. Over the years, the response rate remained high and varied from 93.1% (2012) to 85.5% (2019). Unfortunately, completed data about the general characteristics of stroke services was only partially available in 2012 due to a shortcoming in data collection (***Table 1***).

**Table 1:** Characteristics of the study population in 2012, 2015 and 2019.


	2012	2015	2019

Number of stroke services invited	72	59	55

Number of stroke serviced responded (response rate in %)	67 (93.1)	53 (89.8)	47 (85.5)

Characteristics of regional stroke services networks			

Age, median (n)	9 (41)^1^	12 (53)	15 (46)

– Range	1–16	3–20	1–24

Number of organisations involved, median (n)	5 (40)	6 (53)	7 (47)

– Range	2–19	2–19	2–23

– 0–4 organisations, %	32.5	26.4	23.4

– 5–7 organisations, %	42.0	41.5	31.9

– 8–9 organisations, %	15.5	11.3	21.3

– 10 organisations or more, %	10.0	20.8	23.4

Volume of stroke patients, median (n)	364 (41)	450 (53)	499 (46)

– Min-Max volume	100–1552	79–1650	80–1200

– 1–300, %	34.1	20.8	21.7

– 301–599, %	43.9	56.6	41.3

– 600–899, %	19.6	18.9	15.3

– 900 or more, %	2.4	3.8	21.7

Coordinator of the regional stroke services network			

– Working hours per week, median (n)	8.0 (37)	8.0 (50)	8.0 (39)

– Range hours per week	1–24	1–24	3–24

– 1–4 hours per week, %	27.0	18.0	25.6

– 5–8 hours per week, %	27.1	45.3	38.5

– 9–12 hours per week, %	24.3	17.0	12.8

– 13 hours or more per week, %	21.6	15.1	23.1

– Appointed (% yes)	91.6	100.0	93.5

Working groups (% yes)	86.6	94.3	95.7

Formal agreements on cooperation (% yes)	80.6	86.8	82.4

Regular meetings with steering group (% yes)	77.4	90.6	73.9


^1^ Because no data were collected in 2012, 26 stroke services did not receive questions on characteristics.

### Characteristics of the regional stroke services networks

In 2012, most of the stroke services responding had existed for nine years. The median age of the regional stroke services networks increased proportionally over the years. By 2019, most networks had existed for 15 years. During these years, the number of organisations involved in the networks increased; in 2012, most regional stroke services networks consisted of five organisations and by 2019 this had increased to seven organisations (partly due to the merging of some networks). In addition, the median volume of stroke patients (‘incidence’) in the regional network increased: 364 patients in 2012 to 499 patients in 2019. Although over the years most coordinators were appointed for eight hours per week, the portion of coordinators appointed for more than eight hours per week was less in 2019 (35.1%) compared to 2012 (45.9%). In 2019, all stroke services networks had both a steering group and a working group. Three quarters of the stroke services networks had regular meetings with a steering group (***Table 1***).

### Implementing integrated care activities

The regional stroke services networks implemented most activities in two clusters ‘Interprofessional teamwork’ (3 items) and ‘Roles and tasks’ (8 items) (***Table 2***). In 2019, the networks had implemented 88.7% of the activities for ‘Interprofessional teamwork’ and 85.6% of the activities for ‘Roles and tasks’. Between 2012 and 2019, a statistically significant increase in the mean proportion of activities integrated was found for ‘Transparent entrepreneurship’, ‘Roles and tasks’, ‘Patient-centeredness’ and ‘Commitment’ (‘Transparent entrepreneurship’ (MD = 18.0% F(1) = 10.693, p = 0.001), ‘Roles and tasks’ (MD = 14.0% F(1) = 9.255, p = 0.003), ‘Patient-centeredness’ (MD = 12.9% F(1) = 9.255, p = 0.003), and ‘Commitment’ (MD = 11.2% F(1) = 4.982, p = 0.028). These improvements were mostly made during 2012 and 2015. After 2015, a reduction in implemented activities within the clusters occurred in the networks. This reduction was statistically not significant. ‘Performance management’ decreased by nine percentage points from the average score in 2015 (F(1) = 4.068, p = 0.046).

**Table 2:** Completed activities within the nine clusters of integrated care activities in 2012, 2015 and 2019^1^.


	2012	2015	2019	2012–2015	2015–2019	2012–2019

	mean % (95% CI)	mean % (95% CI)	mean % (95% CI)	mean difference F(df = 1)/p	mean difference F(df = 1)/p	mean difference F(df = 1)/p

	n = 67	n = 53	n = 47	n = 120	n = 100	n = 114

Interprofessional teamwork (3 items)	85.6 (79.7–91.5)	93.1 (88.9–97.3)	88.7 (82.8–94.5)	7.5 F = 3.924 p = 0.050	–4.4 F = 1.581 p = 0.212	3.1 F = 0.518 p = 0.473

Roles and tasks (8 items)	71.6 (65.6–77.7)	88.7 (84.4–92.9)	85.6 (78.8–92.4)	17.1 F = 19.097 p = 0.000	–3.1 F = 0.611 p = 0.436	14.0 F = 9.255 p = 0.003

Patient-centeredness (10 items)	59.0 (53.4–64.7)	71.0 (65.1–76.9)	71.9 (65.7–78.1)	12.0 F = 8.518 p = 0.004	–0.9 F = 0.045 p = 0.842	12.9 F = 9.246 p = 0.003

Commitment (12 items)	58.7 (52.5–65.0)	75.5 (69.5–81.4)	69.9 (61.9–77.8)	16.8 F = 14.515 p = 0.000	–5.6 F = 1.319 p = 0.254	11.2 F = 4.982 p = 0.028

Transparent entrepreneurship (7 items)	49.5 (42.4–56.6)	65.0 (59.0–71.0)	67.2 (59.1–75.2)	15.5 F = 10.375 p = 0.002	–2.2 F = 0.202 p = 0.654	18.0 F = 10.693 p = 0.001

Result-focused learning(14 items)	58.5 (52.2–64.8)	67.1 (61.6–72.6)	67.4 (61.0–73.8)	8.6 F = 3.938 p = 0.050	0.3 F = 0.005 p = 0.945	8.9 F = 3.675 p = 0.058

Quality of care(7 items)	53.5 (48.5–58.6)	66.3 (60.7–71.9)	61.4 (54.5–68.3)	12.8 F = 11.427 p = 0.001	4.9 F = 2.466 p = 0.120	7.9 F = 3.585 p = 0.061

Delivery system (18 items)	54.3 (49.7–59.0)	67.5 (62.1–72.8)	60.3 (53.4–67.2)	13.2 F = 13.962 p = 0.000	–7.2 F = 2.831 p = 0.096	6.0 F = 2.221 p = 0.139

Performance management (18 items)	52.2 (46.5–57.8)	67.1 (61.6–72.6)	58.0 (50.6–65.4)	14.9 F = 13.723 p = 0.000	–9.1 F = 4.068 p = 0.046	5.8 F = 1.596 p = 0.209


^1^ Cluster scores were calculated as proportions of the activities implemented.

Most networks moved on to a later phase of the Development Model for Integrated Care (*X*^2^ (6) = 15.669, p = 0.016, n = 167). In 2019, 71 percent of the networks had reached one of the last two phases (‘Expansion and monitoring phase’ and ‘Consolidation and transformation phase’) (***Table 3***).

**Table 3:** Stroke services per phase of development based on the Development Model for Integrated Care in 2012, 2015 and 2019^1,2^ (in %).


PHASE OF DEVELOPMENT	2012 (N = 67)	2015 (N = 53)	2019 (N = 47)

Initiative and design phase	37.3	34.0	8.5

Experimental and execution phase	9.0	7.5	21.3

Expansion and monitoring phase	35.8	35.8	48.9

Consolidation and transformation phase	17.9	22.6	21.3


^1^ The phase of development is assessed by having implemented at least seven of the top-ten relevant items for a certain phase of development (see Appendix 2). ^2^ Pearson’s *X*^2^ (6) = 15.669, p = 0.016, n = 167.

### Association between characteristics of networks and cluster scores

The exploration of the association between characteristics of stroke services networks and cluster’ scores confirmed a positive correlation between the age of the regional stroke services networks and some clusters (‘Roles and tasks’ (B = 1.387 CI = 0.763–2.011 p = 0.000), ‘Commitment’ (B = 1.625 CI = 0.862–2.388 p = 0.000), ‘Transparent entrepreneurship’ (B = 1.912 CI = 1.152–2.677 p = 0.000), ‘Result-focused learning’ (B = 1.491 CI = 0.784–2.199 p = 0.004), ‘Delivery system’ (B = 1.402 CI = 0.754–2.050 p = 0.000) and ‘Performance management’ (B = 1.495 CI = 0.31–2.159 p = 0.000). Besides, the number of organisations involved is also associated with further implementation on ‘Transparent entrepreneurship’ (B = 1.607 CI = 0.534–2.680 p = 0.004) and ‘Quality of care’ (B = 1.088 CI0.071–2.106 p = 0.036). A small but negative association was found between the volume of patients and performing the activities in two clusters, ‘Interprofessional teamwork’ (B = –0.015, CI = –0.028 to –0.003, p = 0.019) and ‘Patient-centredness’ (B = –0.014, CI = –0.027 to –0.001, p = 0.040) (***Table 4***).

**Table 4:** Regression analysis with forward selection^1^ on completed integrated activities within the nine clusters and characteristics of integrated stroke services within and over the years (B, CI, p).


	YEAR	R^2^	AGE OF REGIONAL STROKE SERVICES NETWORK	ORGANISATIONS INVOLVED	VOLUME OF STROKE PATIENTS, LAST YEAR

Inter-professional teamwork (3 items)	All	0.045			B = –0.015 –0.028 to –0.003 p = 0.019

Roles and tasks (8 items)	2012	0.135	B = 2.074 0.247 to 3.902 p = 0.027		

	2019	0.140	B = 0.896 0.144 to 0.165 p = 0.021		

	All	0.137	B = 1.387 0.763–2.011 p = 0.000		

Patient-centeredness (10 items)	2019	0.118			B = –0.021 –0.041 to –0.002 p = 0.035

	All	0.092	B = 1.005 0.320 to 1.691 p = 0.004		B = –0.014 –0.027 to –0.001 p = 0.040

Commitment (12 items)	2015	0.098			B = 1.709 0.202 to 3.217 p = 0.27

	2019	0.227	B = 1.729 0.652 to 2.806 p = 0.02		

	All	0.160	B = 1.625 0.862 to 2.388 p = 0.00	B = 1.302 0.229 to 2.375 p = 0.018	

Transparent entrepreneurship(7 items)	2012	0.274	B = 2.776 0.656 to 4.897 p = 0.012	B = 3.454 0.605 to 6.304 p = 0.019	

	2015	0.085	B = 1.810 0.684 to 2.935 p = 0.002	B = 1.588 0.080 to 3.096 p = 0.039	

	2019	0.228	B = 1.914 1.152 to 2.677 p = 0.00	B = 1.607 0.534 to 2.680 p = 0.004	

	All	0.213	B = 1.914 1.152 to 2.677 p = 0.00	B = 1.607 0.534 to 2.680 p = 0.004	

Result-focused learning (14 items)	2019	0.173	B = 1.344 0.351 to 2.336 p = 0.009	B = 1.154 0.158 to 2.150 p = 0.024	

	All	0.155	B = 1.491 0.784 to 2.199 p = 0.004		

Quality of care (7 items)	2015	0.111		B = 1.705 0.305 to 3.105 p = 0.018	

	All	0.035		B = 1.088 0.071 to 2.106 p = 0.036	

Delivery system(18 items)	2019	0.204	B = 1.442 0.478 to 2.406 p = 0.004		

	All	0.131	B = 1.402 0.754 to 2.050 p = 0.000		

Performance management (18 items)	2019	0.294	B = 1.754 0.836 to 2.673 p = 0.000		

	All	0.140	B = 1.495 0.831 to 2.159 p = 0.000		


^1^ Criterion: probability-of-F-to-enter <= 0.050. ^2^ Only the statistically significant correlations are presented. No correlations with ‘working hours of the coordinator’ were found.

## Discussion

A unique national self-assessment cohort study among stroke services was conducted to establish which integrated activities were performed by Dutch regional stroke networks in 2012, 2015 and 2019. This first national longitudinal study revealed that stroke services had developed further during this period of time, stroke services implemented statistical significantly more integrated care activities in four out of nine clusters (i.e. transparent entrepreneurship, roles and tasks, patient-centredness, commitment). Networks with more years of experience in collaboration and networks with more stakeholders involved, turned out be further developed in terms of maturity of integrated care. Simultaneously, a higher volume of patients seems to be associated with performing less activities on patient-centredness and interprofessional teamwork.

The results resemble other study findings in studies of integrated care networks for other patient groups [18,19]. Notwithstanding these positive results, integrated stroke care services networks should pay attention to implementing activities that are more linked to continuous quality improvement of care processes and outcomes of care (the ‘Result-focused learning’, ‘Quality of care’, ‘Delivery system’ and ‘Performance management’ clusters). The way activities in integrated care networks should be implemented needs to be tailored. Realistic evaluations paying attention to what works for whom in what circumstances and how may be helpful in this respect [20,21]. Some improvement activities may relate to minor changes in practice, whereas others may reveal larger cultural or organisational issues that need to be addressed and hence have longer timespans [22]. In addition to measuring the level of implementation of integrated activities it is necessary to also measure outcomes of integrated care to understand how outcomes can be improved. This is challenging since, among other things, data collection for further development of integrated care networks is hindered by the use of multiple registries in the organisations involved and the absence of a well-structured approach to systematically collect relevant and meaningful outcome data. A comprehensive quality improvement programme with a focus on continuous evaluation of the context, mechanisms and outcomes of integrated stroke care would be regarded helpful in enabling the provision of patient-centred, integrated care services [23,24].

## Leadership by integrated care network coordinators

A review on leadership in integrated care networks by Mitterlechner [25] underlines the importance of care coordinators in moving a network forward besides other factors such as network governance, trust and organisational structures. Integrated care network coordinators are needed for promoting collaboration in the network by encouraging communication, by creating inter-organisational linkages, by gathering stakeholders for problem solving and by facilitating the involvement of relevant parties. Within the regional networks, coordinators and stakeholders are then still able to evaluate the collaboration, processes and outcomes of integrated stroke care in their networks in a practical and participatory way and support continuous reflective learning [19]. The KNCN learning network gives coordinators of regional stroke services networks the opportunity to ‘teach each other and to learn from each other’ which could also benefit the improvement of integrated care activities and learning between services.

## Scale of the regional stroke services network

After 2015, an apparent reduction in completed activities within the clusters took place in the networks, due to some regional networks merging. A significant decrease in the mean score for ‘Performance management’ was found. The association of integrated care activities with the volume of patients (small and negative) and the number of organisations involved (positive) seems interesting in this respect. Adding more organisations to the networks apparently encourages structured collaboration on integrated care in the region. On the other hand, a higher volume of patients could impede ‘patient-centredness’ and regional networks should consider which patients (or groups of patients) should be their target groups and what is the most suitable scale for stroke services. The relationship between scale and patient-centeredness remains mainly unexplored in integrated care [15]. Patient-centredness in integrated care could include activities concerning the design of processes of care, training of professionals, communication and information exchange and patient’ involvement in care [26].

Since 2018, the Dutch government has been promoting the transfer of care and the collaboration between stakeholders within localities by means of a national programme called ‘The right care, at the right place, at the right time, with the right resources’. This movement favours regional collaboration, and therefore increases the need for effective implementation of integrated stroke care services within the regional networks at both the local and interregional levels especially when shortages of resources (e.g. staff, money) are experienced [15]. Further studies into the relationships of the organisation and scale of integrated care networks with patient-reported experiences and outcomes are needed.

## Strengths and limitations and suggestions for further research

The strengths of the current study are the repeated measurements over a period of seven years by a validated model and the high response rate of stroke services. The DMIC is a model that helps to create an understanding of the presence of the activities as part of the delivery of integrated care services [27]. Although the DMIC was developed in a former decade, the model seems still valid, including the most relevant activities for an integrated care network. The model is still applied in recent studies and considered relevant in a diversity of countries and contexts, including Parkinson Networks in Germany, integrated services networks in Canada and diabetes networks in the Netherlands [19,28,29]. However, this study also has some limitations. First, as this study uses a self-assessment method, the answers given are subjective and open to bias. Coordinators may have had different frames of reference or interpretations of the integrated activities or the level to which they have been implemented. Second, the questionnaires were completed by the coordinator on behalf of the stroke services networks and no data is available about whether and how coordinators involved stakeholders of the regional networks in providing their answers. Thirdly, the DMIC does not involve an assessment of the outcomes of the integrated care services. This is a recommendation for further research. Fourth, the relationships between the characteristics (e.g. scale), development of services and outcomes need to be further addressed in future research. In our study, a regression analysis was used to explore the association between integrated care activities and characteristics of integrated stroke services. A better understanding is needed on the impact of features on performance. Finally, the study results are primarily valid for the Dutch healthcare system. Nonetheless, the Action Plan for Stroke in Europe 2018–2030 formulates targets for integrated stroke care that are in line with the results of our study: 1) someone should be responsible for stroke quality improvement in the region; 2) systems for assessing/accrediting stroke services are needed with peer support for quality improvement; and 3) patient-reported and clinical outcomes covering both hospital and community care should be collected to improve healthcare [30].

## Conclusion

An increase was found in the development of integrated care activities within the regional stroke networks in the Netherlands, from 2012 until 2019. Further, experience in collaboration in the networks benefits the performance of integrated care activities. In the context of the Netherlands, stroke services with longer collaboration timespans do result in improved development phases, as defined by the DMIC, and a more comprehensive range of elements of integrated care. However, after two decades of implementation of stroke services, there is room for further improvement. This illustrates the long-term commitment that is needed if these complex programmes or strategies are to provide added value.

## Data accessibility statement

All data are archived by the first author (HVP).

## Additional files

The additional files for this article can be found as follows:

10.5334/ijic.5611.s1 Appendix 1. Overview Over Integrated Activities Asked for.

10.5334/ijic.5611.s2 Appendix 2. Development Model for Integrated Care – Description of development phases.
