# Supplementary material for: Optimising Integrated Stroke Care in Regional Networks: A Nationwide Self-Assessment Study in 2012, 2015 and 2019
Source: Int J Integr Care. 2021 Sep 20;21(3):12. doi: 10.5334/ijic.5611 (PMC8462476; doi:10.5334/ijic.5611)
Supplement: Appendix 1. — Overview Over Integrated Activities Asked for. [file ijic-21-3-5611-s1.pdf]

APPENDIX 1: OVERVIEW OVER INTEGRATED ACTIVITIES ASKED FOR.

1. Name of the integrated care service:
2. Starting year of the collaboration in the care service: (Year)
3. Number of included patients/clients in the year: (Last total year)
4. Care providers: (tick box: General practitioners, Hospital, Rehabilitation clinics, Nursing homes, Homes for the elderly, Home care organisations, Mental health care, Welfare organizations, Municipalities, other.
5. Total number of involved health care organizations:
6. Collaboration agreements with the general practitioners: (yes/no)
7. Collaboration agreements with ambulatory care services: (yes/no)
8. Are there periodically meetings regarding this integrated care service with (tick box: Health care insurers, Assessment agencies, Municipalities, Client federations, None of these)
9. Are there on an operational level working groups which focus on the improvement and development of the integrated care service: YES/NO. If YES: Who participates in these working groups? (tick box: Practitioners, Managers, Both)
10. Is there a coordinator on the level of the integrated care service which has coordinating tasks? YES/NO If YES: how many hours per week does this person have for these tasks: (hours/week)
11. Is there a formal collaboration agreement signed up by the CEO's of the health care organizations? (yes/no)
12. Are there regular meetings on CEO level/a steering committee regarding this integrated care service? (yes/no)
13. Elements of integrated care within the nine clusters are presented. (tick box: relevant, is present yes, since (year), not present, planned).

C1 Interprofessional teamwork	
1	Defining the targeted client group
2	Working in multidisciplinary teams
3	Reaching agreements on the availability and accessibility of professionals

C2 Roles and tasks<br>4 Directing the care chain by appointing a limited number of people with coordinating tasks<br>5 Installing a coordinator working at the chain-care level<br>6 Reaching agreements among care partners on tasks, responsibilities and authorizations<br>7 Establishing the roles and tasks of multidisciplinary team members<br>8 Ensuring that professionals in the care chain are informed of one another's expertise and tasks<br>9 Achieving adjustments among care partners by means of direct contact<br>10 Realizing direct contact among professionals in the care chain<br>11 Reaching agreements on introducing and integrating new partners in the care chain
C3 Patient-centeredness<br>12 Developing a front office: single entry point for client information<br>13 Collaboratively offering client information of the care partners<br>14 Providing understandable and patient-centred information<br>15 Using self-management support methods as a part of integrated care<br>16 Implementing care process-supporting clinical information systems<br>17 Developing care programmes for relevant client subgroups<br>18 Flexible adjustment of integrated care corresponding to individual clients' needs<br>19 Designing care for clients with multi- or co-morbidities<br>20 Using a protocol for the systematic follow-up of clients<br>21 Monitoring client judgements and satisfaction for the whole care chain
C4 Chain Commitment<br>22 Defining the ambitions and aims of the collaboration in the care chain<br>23 Signing collaboration agreements among the care partners<br>24 Establishing dependencies among care partners<br>25 Guiding the care chain by emphasizing a collaborative commitment<br>26 Assuring the leadership commitment of the partners involved in the care chain<br>27 Stimulating the awareness of working in a care chain

28 Structural meetings of the leaders of the care-chain organizations<br>29 Structural meetings with external parties, such as insurers, local governments and inspectorates<br>30 Describing the tasks and authorities of leaders, coordinators and advisory boards in the care chain<br>31 Stimulating trust among care partners<br>32 Reaching agreements about letting go care partner domains<br>33 Attention to connectivity with housing, social care and social services throughout the care chain
C5 Transparent Entrepreneurship<br>34 Making a commitment to a joint responsibility for the final goals and results to be achieved<br>35 Reaching agreements on the financial budget for integrated care<br>36 Allocating financial budgets for the implementation and maintenance of integrated care<br>37 Offering a single collaborative financial contract to the financing parties through the collective of care partners<br>38 Creating an open environment that encourages experiments and pilot projects<br>39 Involving leaders in improvement efforts in the care chain<br>40 Using a uniform language in the care chain
C6 Results focused learning<br>41 Defining and assessing the characteristics of the collaboratively delivered care<br>42 Making the benefits of the collaboration transparent for each care-chain partner<br>43 Linking consequences to the achievement of goals agreed upon<br>44 Integrating incentives for rewarding the achievement of quality targets<br>45 Using knowledge and information for directing and coordinating the care chain<br>46 Sharing knowledge among care partners about effectively organizing sustainable integrated care<br>47 Collaboratively assessing bottlenecks and gaps in care<br>48 Collaborative learning in the care chain in order to innovate integrated care

49 Striving toward an open culture for discussing possible improvements for care partners<br>50 Stimulating a learning culture and continuous improvement in the care chain<br>51 Learning by the exchange of information among professionals about the care process<br>52 Introducing collaborative education programmes and learning environments for the care professionals<br>53 Reaching agreements on required levels of expertise at the stroke units<br>54 Reaching agreements on required levels of expertise at rehabilitation
C7 Quality of care<br>55 Systematically assessing the needs of the clients in the care chain<br>56 Developing a multidisciplinary care pathway<br>57 Using evidence-based guidelines and standards<br>58 Involving client representatives in monitoring the performance of the care chain<br>59 Involving client representatives in improvement projects in the care chain<br>60 Providing patients an aftercare process (after the clinical and rehabilitation phase) with a clear point of contact.<br>61 Organising a 24-hour availability (7 days a week) of thrombolysis in the care chain
C8 Delivery system<br>62 Reaching agreements on chain logistics (e.g. waiting periods and throughput times)<br>63 Reaching agreements on referrals and the transfer of clients through the care chain<br>64 Developing criteria for the inclusion and throughput of clients in the care chain<br>65 Reaching agreements on linking clients to outside resources or community care partners<br>66 Reaching agreements among care partners on scheduling client examinations and treatment<br>67 Developing criteria for assessing clients' urgency<br>68 Reaching agreements among care partners on discharge planning<br>69 Reaching agreements among care partners on managing client preferences<br>70 Reaching agreements among care partners on providing care to waiting-list clients<br>71 Reaching agreements on procedures for information exchange<br>72 Reaching agreements on procedures for the exchange of client information

73 Using uniform client-identification numbers within the care chain<br><br>74 Developing connections with the databases of partners in the care chain<br><br>75 Using shared client treatment and care plans<br><br>76 Using a single client-monitoring record accessible to all care partners<br><br>77 Reaching agreements among care partners on the consultation of experts and professionals<br><br>78 Deploying specialized nurses within the care chain<br><br>79 Offering case management to clients with complex needs
C9 Performance management<br><br>80 Defining performance indicators to evaluate the results of the integrated care delivered<br><br>81 Establishing quality targets for the performance of the whole care chain<br><br>82 Establishing quality targets for the performance of care partners<br><br>83 Gathering data on client logistics (e.g. volumes, waiting periods and throughput times) in the care chain<br><br>84 Gathering client-related performance data (health status, quality of life)<br><br>85 Measuring patient satisfaction throughout the whole care chain<br><br>86 Gathering financial performance data of the care chain<br><br>87 Making transparent the effects of the collaboration on the production of the care partners<br><br>88 Reaching agreements about the uniform use of performance indicators in the care chain<br><br>89 Using a systematic procedure for the evaluation of agreements, approaches and results<br><br>90 Monitoring and analysing mistakes/near mistakes in the care chain<br><br>91 Monitoring successes and results during the development of the integrated care chain<br><br>92 Installing improvement teams at the care-chain level<br><br>93 Providing feedback to care partners on transfers<br><br>94 Using feedback and reminders by professionals for improving care<br><br>95 Monitoring whether the care delivered corresponds with the evidence-based guidelines<br><br>96 Sharing the benchmark results within the care chain for the purpose of improving the care chain<br><br>97 Identifying performance indicators to evaluate the results of integrated care
