# Supplementary material for: Optimising Integrated Stroke Care in Regional Networks: A Nationwide Self-Assessment Study in 2012, 2015 and 2019
Source: Int J Integr Care. 2021 Sep 20;21(3):12. doi: 10.5334/ijic.5611 (PMC8462476; doi:10.5334/ijic.5611)
Supplement: Appendix 2. — Development Model for Integrated Care – Description of development phases. [file ijic-21-3-5611-s2.pdf]

APPENDIX 2 Development Model for Integrated Care – Description of development phases.

PHASE 1 Initiative and design phase

The collaboration between health care providers has been intensified or started up. The starting point is a common problem or chance occurrence, or builds on current cooperation among care professionals. There is a sense of urgency and there are possibilities for working on these challenges in collaboration. The targeted patient group, the care chain and care process have been defined, as also the needs of patients and stakeholders. The level of ambitions, motivation and leadership determine the progress achieved. A multidisciplinary team designs an experiment or project to execute the current ideas. The collaboration can be signed up to in an agreement among care partners. Keywords: Exploring possibilities/impossibilities, ambitions and chances, (project) design and collaboration agreements. [Items 22, 1, 41, 39, 34, 23, 30, 63, 23, 72 on the list of integrated activities in Appendix 1]

PHASE 2 Experimental and execution phase

New initiatives or projects are being executed in the care chain. The aims, content, roles, and tasks in the care chain have been clarified and written down in care pathways and protocols. There is coordination at the level of the care chain by for instance installing coordinators or setting up meetings. Information about patient groups, working procedures or professional knowledge is exchanged. There are experiments within the collaboration, results are evaluated to learn from and reflect on. Preconditions for projects have been considered and boundary conditions have been solved by collaborative means or agreements among care providers. Key words: Writing down aims and content of the collaboration, coordination at care chain level, experimenting and reflecting. [Items 10, 75, 78, 9, 57, 91, 68, 2, 8, 83 on the list of integrated activities in Appendix 1]

PHASE 3 Expansion and monitoring phase

Projects have been expanded or integrated in integrated care programs. Agreements on the content, tasks and roles within the care chain are clear and signed up. Collaboration is no longer on an informal basis. Results are systematically monitored and improvement areas identified. The targeted population has

been surveyed. More collaborative initiatives emerge such as mutual education programs. There is a continuous commitment to the ambition of the integrated care program. Interorganisational barriers and fragmented financial structures are on the agenda of the care partners. Keywords: Further development and maturity, monitoring and improving results, new questions and innovation.

[Items 89, 18, 90, 11, 52, 59, 19, 48, 74, 87 on the list of integrated activities in Appendix 1]

PHASE 4 Consolidation and transformation phase

The integrated care program is the regular way of working and providing care. Coordination at care chain level is operational; information is shared, transferred and fed back. A monitoring system periodically shows if results are being sustained, what specific improvement possibilities have been identified and to what extent patient needs have been met. The program builds further on successful results. Organisational structures transform or are newly designed around the integrated care program. Financial agreements are arranged with financers by means of integral contracts covering the care chain as a whole. Partners in the care chain explore new options for collaboration in the external environment with other partners. Keywords: Continuous improvement, new ambitions, structures fitting the integrated care program (organisational structures, integral financing).

[Items 37, 43, 44, 29, 46, 52, 90, 17, 32, 35 on the list of integrated activities in Appendix 1]
